# InChI version 1.06: now more than 99.99% reliable

**DOI:** 10.1186/s13321-021-00517-z

**Published:** 2021-05-24

**Authors:** Jonathan M. Goodman, Igor Pletnev, Paul Thiessen, Evan Bolton, Stephen R. Heller

**Affiliations:** 1 Centre for Molecular Informatics, Yusuf Hamied Department of Chemistry, Lensfield Road, Cambridge, CB2 1EW UK; 2 InChI Trust, Cambridge, UK; 3 Department of Chemistry, Lomonosov Moscow State University, Moscow, 119991 Russia; 4 National Center for Biotechnology Information, National Library of Medicine, National Institutes of Health, Bethesda, MD 20894 USA

**Keywords:** InChI, InChIKey, PubChem, RInChI

## Abstract

The software for the IUPAC Chemical Identifier, InChI, is extraordinarily reliable. It has been tested on large databases around the world, and has proved itself to be an essential tool in the handling and integration of large chemical databases. InChI version 1.05 was released in January 2017 and version 1.06 in December 2020. In this paper, we report on the current state of the InChI Software, the details of the improvements in the v1.06 release, and the results of a test of the InChI run on PubChem, a database of more than a hundred million molecules. The upgrade introduces significant new features, including support for pseudo-element atoms and an improved description of polymers. We expect that few, if any, applications using the standard InChI will need to change as a result of the changes in version 1.06. Numerical instability was discovered for 0.002% of this database, and a small number of other molecules were discovered for which the algorithm did not run smoothly. On the basis of PubChem data, we can demonstrate that InChI version 1.05 was 99.996% accurate, and InChI version 1.06 represents a step closer to perfection. Finally, we look forward to future releases and extensions for the InChI Chemical identifier.

## Introduction

The first version of InChI was made publicly available in the spring of 2005 and further versions [1–5], including a separate InChI for Reactions (RInChI [6, 7]), have been released over the years. The original version covered much of organic chemistry and it was quickly adopted and used by chemists and scientific databases throughout the world. Its application in virtual databases includes SAVI-2020, a database of reactions, labelled by RInChI, with over 1.75 billion products, all of which are assigned InChI [8]. The effective coverage of chemistry had a downside: the pressure to extend and add features and capabilities was rather small. The InChI software, coded in the C language, contains about 200,000 lines of code and comments. Such a large computer program will always have bugs and imperfections. We are very grateful to everyone that has reported issues and look forward to more feedback from the large and growing community of InChI users.

The InChI is canonical. This central feature of the design requires that for every molecule there is just one InChI, and every InChI identifies just one molecule. This makes it possible for the InChI to be used as an identifier to link identical structures in databases or on the internet. It has been and is being used by many commercial and non-commercial software chemistry packages. The InChI fulfils a different role to SMILES [9], which was designed to be easily understood both by chemists and by computers. As a consequence, there are many valid SMILES representations of every molecule. This distinction makes SMILES a better tool for some applications than InChI, and InChI a better tool for others, whenever a unique identifier is helpful. There are a variety of schemes to generate canonical SMILES, but just one InChI algorithm.

**Fig. 1:**
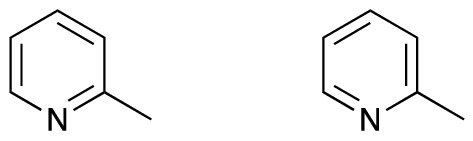
These two molecules are the same as each other, and should have the same name

The issue is more subtle than simply finding a globally-agreed canonicalization algorithm. It is also necessary to address the fundamental question: what is a molecule? InChI provides a consistent and effective answer to this question, which covers nearly all of the molecules used in medicinal chemistry. SMILES has the option of encoding molecular detail that InChI does not, which makes it more flexible in its descriptions of complex objects, and the disadvantage that an object that some people consider to be one molecular system is considered as several different molecular systems by other people, with different SMILES strings. For example, 2-methylpyridine, which is also known as picoline, can be drawn in two different ways with different double bond positions (Fig. 1). Both of these representations are correct. There is just one InChI for both forms: InChI = 1 S/C6H7N/c1-6-4-2-3-5-7-6/h2-5 H,1H3. There are many valid SMILES, including CC1=CC=CC=N1 and CC1=NC=CC=C1.

The SMILES contain more information, because they record the localisation of the double bonds and the InChI does not. The localisation can lead to problems in assigning stereochemistry using the Cahn-Ingold-Prelog rules [10]. In this particular example, the extra information does not correspond to any physically observable distinction for the molecule these strings represent. Might there be molecules for which a different InChI is applied to variant representations of the same molecule, or else an important distinction in structure is missed in InChI generation? The choices that the InChI algorithm makes about what is and is not represented control how effective it is at producing useful molecular identifiers.

Tautomers represent one aspect of this question. Are two tautomers the same molecule or different molecules? There are many cases for which the answer depends on the detail of the question. Tautomers may not be separable when stored in a bottle in a storeroom but the distinction between them may be important in understanding a reaction mechanism, during which they may be formed transiently, or at a very low temperature. Should they have the same InChI or different InChI? A detailed investigation of this issue is underway [11] and is beyond the scope of this paper. A few molecular features, such as atropisomerism and some aspects of organometallic structures, are not described by the current version of the InChI. Work is underway to introduce these features into future releases of the InChI.

In this manuscript we report the details of the latest upgrade, version 1.06, which fixes a series of known issues, and the results of its extensive testing on the PubChem Compound database provided by the U.S. National Center for Biotechnology Information, containing more than one hundred million structures [12]. For a program to be considered reliable, its outputs must be consistent, reproducible, and widely-applicable. The program must be easy to use. We investigate all of these aspects of reliability.

### Results: details of upgrade

InChI v1.06 is available for download from the InChI Trust website [13]. In addition to the software and its source code, the downloads include a detailed log of changes, an API reference, and a list of known issues. Comments are welcomed on the SourceForge discussion list [14].

### Upgrade to v1.05

The key features introduced in v1.05 were the ability to treat polymers and the ability to generate InChI for large molecules containing up to 32,767 atoms. These are still considered to be experimental features. For this reason, the InChI generated for polymers and for molecules with more than 1024 atoms are not standard InChI (prefix InChI = 1 S) but are marked as beta non-standard InChI, indicated by the InChI = 1B prefix. A partial ability to use Molfile v3000 as input format was also added to v1.05. This facility is important in handling large molecules as those in the Molfile v2000 format cannot contain more than 999 atoms.

### Upgrade to v1.06

The upgrade to v1.06 includes support for pseudo-element atoms, labelled “Zz” or “*”, and improved support for single-strand polymers options. All of these facilities are currently only available in the non-standard InChI, labelled with the prefix InChI = 1B. In addition, there are a variety of bugfixes, updates to the API library, support for Intel Threading Building Blocks (TBB) scalable memory allocators and several convenience features.

Support for single-strand polymers was first introduced in v1.05 and has been extended and improved in v1.06. Both structure-based and source-based representation and encoding of polymers are available. Source-based representation encodes chemical structures of the starting material(s) with an indication of polymer nature, type of polymer (block, random, and alternating) and the role and order of the components where needed. Structure-based representation of polymers is based on the structure of structural repeating units, SRU, sometimes called constitutional repeating units, CRU, enclosed in polymer brackets. Note that source- and structure-based representations and their InChI encodings are independent and, in general, no procedures are implied for algorithmic conversion and relation from one type to the other.

A pseudo-element atom is a generic placeholder designating an undefined, unknown or variable entity, and is indicated by “Zz” or “*”. The exact meaning is not defined within the InChI standard. It may be used to indicate the places where constitutional repeat units in polymers join on to each other, or, more generally, to indicate undefined univalent atoms. Stereochemistry adjacent to Zz atoms is disabled by default, although an option is provided to switch it on. This feature makes it possible to describe molecular systems more flexibly, but it may be harder to describe them canonically. The central importance of canonical representation for the InChI is the reason that these features are not a part of the standard InChI (prefix InChI = 1 S) and restricted to the non-standard InChI (prefix InChI = 1B).

**Fig. 2:**
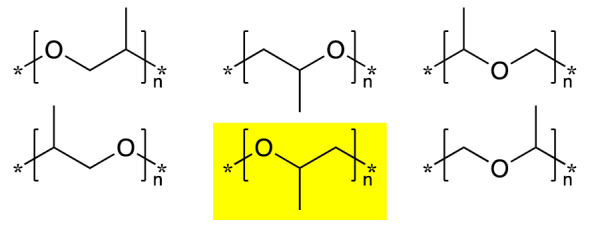
Polypropylene glycol can be described with any of these monomers. InChI version 1.06 selects the highlighted one as the canonical version

This new feature improves the ability of InChI to describe polymers. For example, polypropylene glycol (PPG, [-O-CH2-CH(CH3)-]n) can be described, in structure-based representation, in several different but equivalent ways, Fig. 2.

All six of these representations, which use a * to indicate the Zz atom, are correct; the InChI algorithm selects the highlighted one, in the middle of the bottom row, as the canonical one: InChI = 1B/C3H6OZz2/c1-3(2-5)4-6/h3H,2H2,1H3/z101-1-4(6 -4, 5-2).

Note that the canonical form here is exactly the same as one preferred by IUPAC recommendations [15]. Special care has been taken in developing the InChI algorithm to ensure that the basic IUPAC criteria are incorporated, but the correspondence between the preferred InChI and the IUPAC recommendation (targeted to manual selection of preferred form) is not always perfect.

The InChI v1.05 preferred form for this polymer was different as the two Zz pseudo-element atoms, numbered 5 and 6 in this new InChI, were not available. The InChI v1.06 code can generate the older form of the polymer InChI using the Polymers105 option, but its use is deprecated.

### Pseudo-element atoms

**Fig. 3:**
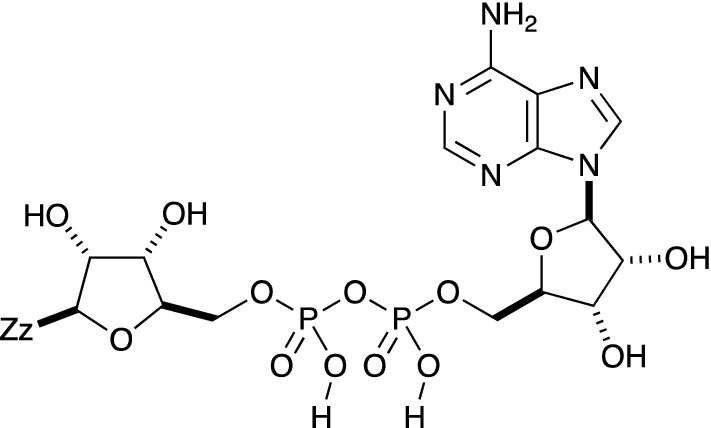
InChI=1B/C15H22N5O13P2Zz/c16-12-7-13(18-3-17-12)20(4-19-7)14-10(23)8(21)5(31- 14)1-29-34(25,26)33-35(27,28)30-2-6-9(22)11(24)15(36)32-6/h3-6,8-11,14-15,21- 24H,1-2H2,(H,25,26)(H,27,28)(H2,16,17,18)/t5-,6-,8-,9-,10-,11-,14-/m1/s1

Pseudo-element atom use is not restricted to polymers. For example, Fig. 3, the adenosinediphosphoribosyl group is a molecular fragment, included in the EBI’s directory of Chemical Entities of Biological Interest (ChEBI) as structure CHEBI:22,259. InChI cannot describe molecular fragments, but the Zz atom in v1.06 provides a non-standard-InChI for this.

### Structure-based issues

**Fig. 4:**
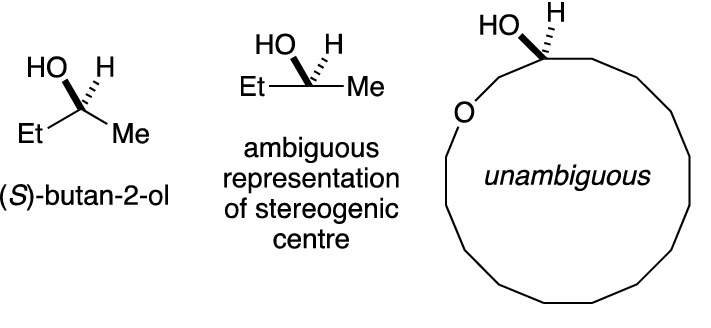
Version 1.06 recognises the large ring and so assigns stereochemistry correctly to the right-hand structure

Translation, from two-dimensional representations of molecules in Molfiles to an InChI, requires interpretation. The InChI code is very effective at doing this, and version 1.06 contains a few improvements. One of these focusses on the representation of large rings, which are used in important classes of molecules including peptides and macrolides. In Fig. 4, the left-hand structure is a clear representation of (S)-butan-2-ol. The stereochemistry of the middle structure is unclear, because the ethyl and methyl groups are represented in a straight line. The InChI code will not give a stereochemical assignment to such a structure. How straight does a line have to be, before giving a stereochemical assignment is inappropriate? The right-hand structure illustrates a molecule for which the stereochemistry can be easily interpreted, because of the large ring, and yet the chain is almost straight. In version 1.06, the InChI code allows for representations of this type to be interpreted by introducing a new option: *LooseTSACheck*. Because this option is for structure perception and not InChI generation, it can be used within the standard InChI.

**Fig. 5:**
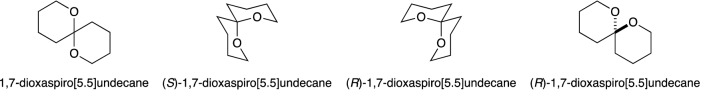
With version 1.05, the InChI of both *R* and *S* 1,7-dioxaspiro[5.5]undecane is InChI = 1 S/C9H16O2/c1-3-7-10-9(5-1)6-2-4-8-11-9/h1-8H2. With version 1.06, a stereochemistry layer is added, and InChI = 1 S/C9H16O2/c1-3-7-10-9(5 -1)6-2-4-8-11-9/h1-8H2/t9-/m1/s1 is generated from the right-hand structure

The InChI software is now able to identify and assign the stereochemistry of 1,7-dioxaspiro[5.5]undecane and related structures (Fig. 5). *R* and *S* 1,7-dioxaspiro[5.5]undecane are mirror images of each other, although this is not immediately obvious from the diagram. InChI v1.06 can now take structures drawn in the form on the right of the figure, and assign the correct stereochemical layer.

Two cases for which version 1.05 was unable to generate an InChI string have been fixed, as have some renumbering issues. The details are given in the description of tests with PubChem, below. Some large molecules supplied in Molfile V3000 formats caused a problem which has now been fixed, as have minor issues in AuxInfo and the non-standard InChI description of polymers.

### Other improvements

Memory allocation can become a serious performance bottleneck when using the InChI library in multi-threading environment, as threads may compete for a global lock related to a single global heap. In this situation, the program’s behaviour is not scalable and speed may degrade if the number of processor cores increases. Intel TBB is free software package available for both Windows and Linux, licensed under the Apache License, which automatically replaces C functions for dynamic memory allocation with its own scalable memory allocators to avoid contention in most cases [16]. This method may optionally be used with the InChI Software library and, in some cases, may provide performance gains.

A new switch, *WMnumber*, has been added which sets the InChI calculation timeout in milliseconds. This complements *Wnumber* which sets the value in seconds. The finer granularity is useful for managing long runs on datasets containing many millions of molecules.

### Security issues

The InChI software expects valid InChI strings or Molfiles as its input. Other input should be rejected with a suitable diagnostic message. A small number of cases have been discovered for which other input files (mostly artificially corrupted) can lead to memory corruption or a crash and there is a possibility that this could cause problems for some applications. A list of people who have pointed out specific issues is in the acknowledgements section of this paper. Numerous modifications to the code have been made to catch these problems.

## Results: tests on PubChem

The InChI algorithm is intended to work on all molecules. Demonstrating that it does this, however, is a non-trivial task. How well does it work on molecules that are not merely un-made, but also un-imagined? A step towards answering this question is to test the algorithm on a large, curated dataset. PubChem [12] is such a database and contained 111 476 790 molecules at the time of testing. Standard InChI strings have been generated for all of these molecules.

Several types of imperfection can be envisaged: (i) failure to create an InChI string; (ii) making the same InChI string for two different molecules; (iii) generating two different InChI strings for the same molecule; (iv) different InChI strings for v1.05 and v1.06.

**Fig. 6:**
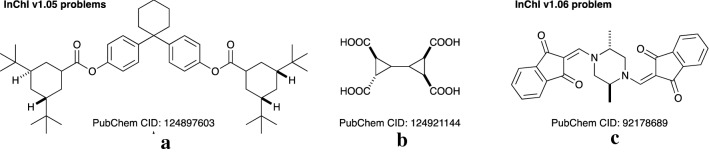
Problem structures from PubChem for InChI v1.05 and InChI v1.06

The first issue, failure to create an InChI string, is serious, but easy to catch because an error message is generated. This problem occurs for two structures for InChI v1.05 and one structure for InChI v1.06 (Fig. 6). The first two, **A** and **B**, both have an undefined stereocentre, and InChI v1.05 gives a STEREOCOUNT_ERR error and does not generate an InChI. The enantiomer of **B** is also present in PubChem (CID: 92,286,308) and InChI v1.05 is able to generate an InChI for this. InChI v1.06 generates InChI strings which note, correctly, that one of the stereocentres is undefined in each case:

**A:** InChI = 1 S/C48H72O4/c1-44(2,3)36-26-32(27-37(30-36)45(4,5)6)42(49)51-40-20-16-34(17-21-40)48(24-14-13-15-25-48)35-18-22-41(23-19-35)52-43(50)33-28-38(46(7,8)9)31-39(29-33)47(10,11)12/h16-23,32-33,36-39 H,13-15,24-31H2,1-12H3/t32?,36-,37+,38-,39-/m0/s1.

**B:** InChI = 1 S/C10H10O8/c11-7(12)3 -1(4(3)8(13)14)2-5(9(15)16)6(2)10(17)18/h1-6 H,(H,11,12)(H,13,14)(H,15,16)(H,17,18)/t1?,3-,4+,5-,6-/m0/s1.

Neither version has any difficulty with the stereoisomers for which all the stereocentres are defined, and both produce the same InChI strings. For molecule **C** (PubChem CID: 92,178,689), InChI v1.05 generates an InChI string, and InChI v1.06 may stop with a CANON_ERR (this appears only in some environments, e.g., Windows/inchi-1 executable but not Linux-64bit/libinchi.so library; the behaviour is expected to be fixed in the next release). There appear to be no other errors of this type for PubChem molecules, and so InChI v1.06 has an error rate of about one in 111 476 790, which can be expressed as 99.999999 % reliable.

The second issue, which would clearly be an error, is generating the same InChI string for two different molecules. When two molecules differ only by the localisation of protons on heteroatoms, they may have the same InChI and would be expected to be inseparable. With these exceptions, no such errors were observed in this survey of PubChem.

**Fig. 7:**
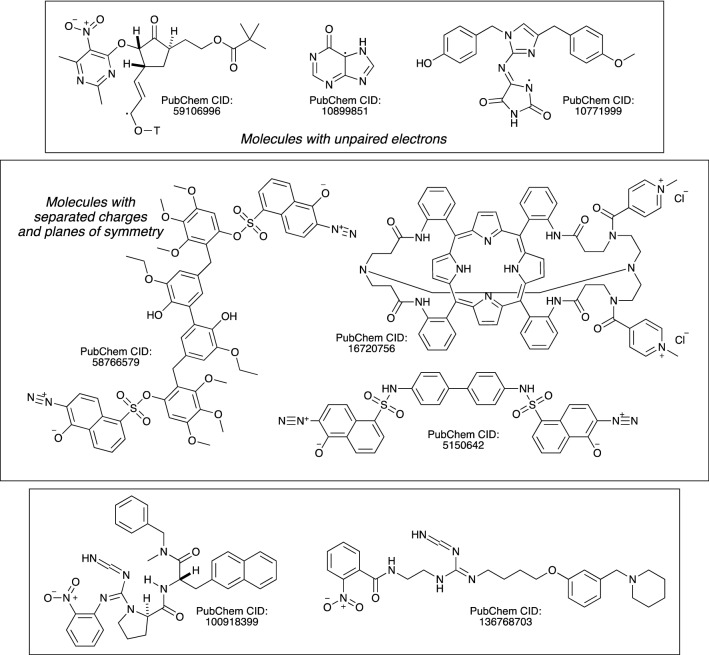
All molecules with numbering instability that lack “/p” and “/q” layers

The third issue, two different InChI strings for one molecule, is also an error. This was observed as a consequence of numbering instability. A key feature of the InChI software is that it numbers all of the atoms in a molecule, except for the hydrogens. This numbering should be the same whatever the order of the atoms in the input Molfile. A molecule with N non-hydrogen atoms can be represented in a Molfile in about N! different ways before the coordinates are considered. All of these possibilities should produce the same InChI string, and they nearly always do. This issue was addressed by using sixteen randomly-generated re-numberings for all the molecules in the database. In nearly all cases, this generated the same InChI string sixteen times. However, 547 examples have been discovered in PubChem for which the numbering of the InChI string can be changed by altering the order of the atoms in the Molfile. Most of these were found with only five renumberings, and only eleven cases were hidden after ten renumberings. These are all listed in the *KnownIssues* file which is a part of the InChI v1.06 download. Nearly all of these molecules contain nitrogen (530 out of 547) and many contain sulfur (453 out of 547), but only 36 contain phosphorous. The majority of these molecules have a net charge (381 positive; 49 negative). Many contain metals (Na: 57; Zn: 6; Ru: 6; K: 4; Fe: 3; Co: 1; Mn: 1; Ni: 1) and come contain halogens (F: 54; Cl: 92; Br: 1; I: 3). Only 84 of the 547 molecules were neutral and did not contain metals; all of these molecules contain nitrogen and three have unpaired electrons. The most distinctive thing about this list of 547 molecules is that 534 of them contain a “/p” layer, indicating that a proton has to be added or removed from the formula to give the input composition. Of the remaining 13 molecules, five have a “/q” layer, indicating a charge on the molecule. This leaves eight, which are illustrated in Fig. 7. With a problem that affects 547 molecules out of 111 476 790, InChI v1.06 is 99.9995% reliable.

**Fig. 8:**
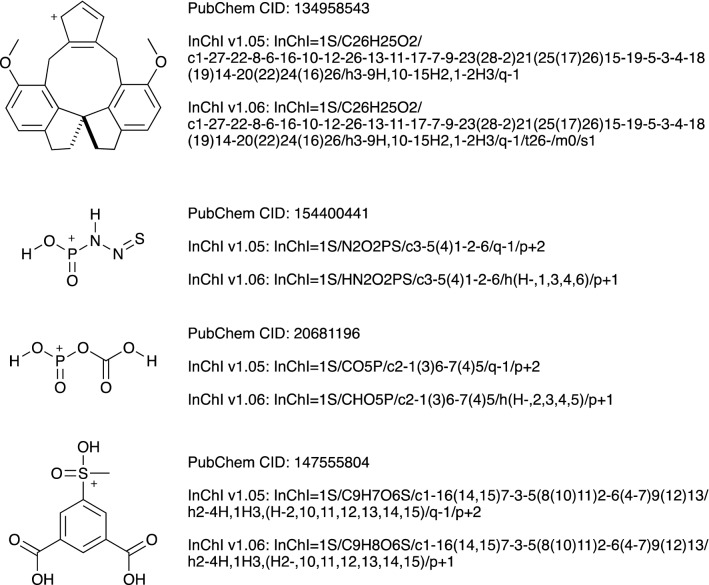
Changes from v1.05 to v1.06

The final issue, when v1.05 and v1.06 produce different strings, may be a result of improved structural perception for v1.06, but may also be inconvenient as molecules that are the same are associated with two different InChI strings, one for v1.05 and another for v1.06. There are 6524 examples of this in PubChem, none of which show numbering instability. Three of these contain just carbon, hydrogen and oxygen; two of these three are Fig. 6a and b. The third is illustrated at the top of Fig. 8. This structure has a stereocentre in a spiro-cyclic motif, which is missed by v1.05 and found by v1.06. The InChI are identical except that v1.06 adds the stereochemical layers: /t26-/m0/s1. In contrast to molecules with unstable numbering, none of the 6524 molecules in this group have a net charge indicated in the molecular formula. However, almost all of them (6388) have a “/p” layer when calculated with InChI v1.06, and 434 have a “/q” layer. 364 have both “/p” and “/q”. This contrasts with the InChI v1.05 calculations on this group, for which 6169 have a “/p” layer, 6323 have a “/q” layer, and 6154 have both “/p” and “/q”. Most of these cases are the result of a fix to a problem with molecules with acidic hydroxy groups at cationic heteroatom centres, which led to issues with numbering atoms. The details of this are in the CHANGELOG file in the release. In v1.05, such systems were often labelled with both a “/q” and a “/p” layer: “/q-1/p + 2” was very common, and occurs in 5446 of the v1.05 InChI strings in this group of 6524 molecules. In v1.06, the two layers are replaced by one layer: “/p + 1” and the extra hydrogen atom is included in the formula of the molecule. Three examples are in Fig. 8.

Only 35 molecules out of 6524 have the same “/q” layer for both InChI v1.05 and InChI v1.06. In 18 of these cases, the match arises because of the absence of a “/q” layer in both versions of the InChI string. In all 35 of these cases, the difference between v1.05 and v1.06 arises in the “/t” (stereochemistry) layer. For PubChem CID: 6,555,836, 6,589,644, 6,589,645, 11,871,423, 18,805,145, 18,805,148, 18,805,149, 18,805,151, 18,805,146, 49,950,537, 49,950,540, 101,988,808 the stereochemistry is not clear in PubChem. This illustrates the power of the InChI string to highlight molecules within a database that would benefit from further checking. These checks demonstrate that the transition from v1.05 to v1.06 is 99.99% consistent for the standard InChI string.

## Discussion

InChI v1.06 provides substantial new functionality and corrects some issues with earlier releases, whilst retaining a very high level of backwards compatibility. Changes to the InChI can be readily incorporated in Reaction InChI (RInChI) [6, 7] and into ongoing work to develop an InChI-based description of mixtures [17].

The frequency of the errors we have discovered in this survey of PubChem can be compared with the number of InChIKey collisions. It was shown by one of us (JMG, [18, 19]) that InChIKey collisions can occur. The paper [20] analyzes the situation in details and demonstrates that collisions, though unavoidable, occur at a very small and expected rate. The probability of an InChIKey collision in a database of the current size of PubChem is slightly above 0.01%. The fact that InChIKey collisions can occur is important to consider, even though their occurrence is so rare that they have been found only by deliberate searching. The observed failure rate of the InChI algorithm of one structure in 111 476 790 is slightly higher than this. The possibility of InChIKey collisions should not be ignored in databases of this size.

## Conclusions

That latest version of the InChI code, version 1.06, provides a significant advance in functionality and reliability, whilst retaining compatibility with previous releases. The upgraded InChI can be used now both as a molecular identifier and as a part of the Reaction InChI (RInChI) process for labelling reactions [6, 7]. The InChI Trust [21] and the IUPAC InChI subcommittee [22] are always pleased to hear suggestions for new functionality and reports of issues with the current code. These should be reported either to the InChI SourceForge website and discussion list [14], or by e-mail to the secretary of the IUPAC InChI subcommittee (Jonathan Goodman, jonathan@inchi-trust.org).

We continue to work on expanding the capabilities of InChI. Organometallics and extended stereochemistry are the likely next additions to InChI capabilities. There are two fundamental issues with further InChI developments. First there is the human element with diverse opinions on what constitutes the correct answer in representing molecular structures. The second is the chemistry itself, which is not always well resolved and unambiguous. Like the InChI capabilities in the area of polymers, where the properties of a polymer are often of more concern to the chemist than the structure, which may not be known in detail. InChI is and always will be limited by how good chemists and chemistry are at representing a structure. The InChI Trust is also developing the functionality of the InChI beyond the representation of single molecules: Reactions, mixtures, Markush structures, QR codes for InChI, and other areas are all being developed and will be made available as soon as possible.

We hope that InChI v1.06 will be widely used. It retains compatibility with previous standard InChI releases, provides additional functionality, and is, on the basis of tests with PubChem, more than 99.99% reliable.

## Data Availability

The InChI v1.06 software is available for download from the InChI Trust website [21].
